# Psychometric properties of an Arabic translation of the external and internal shame scale (EISS)

**DOI:** 10.1186/s12888-023-04729-5

**Published:** 2023-04-11

**Authors:** Feten Fekih-Romdhane, Diana Malaeb, Mariam Dabbous, Rabih Hallit, Sahar Obeid, Souheil Hallit

**Affiliations:** 1 The Tunisian Center of Early Intervention in Psychosis, Department of Psychiatry “Ibn Omrane”, Razi hospital, Manouba, 2010 Tunisia; 2 Faculty of Medicine of Tunis, Tunis El Manar University, Tunis, Tunisia; 3 College of Pharmacy, Gulf Medical University, Ajman, United Arab Emirates; 4 School of Pharmacy, Lebanese International University, Beirut, Lebanon; 5 School of Medicine and Medical Sciences, Holy Spirit University of Kaslik, P.O. Box 446, Jounieh, Lebanon; 6 Department of Infectious Disease, Bellevue Medical Center, Mansourieh, Lebanon; 7 Department of Infectious Disease, Notre Dame des Secours University Hospital Center, Street 93, Byblos, Postal Code 3, Lebanon; 8 Social and Education Sciences Department, School of Arts and Sciences, Lebanese American University, Jbeil, Lebanon; 9 Psychology Department, College of Humanities, Effat University, Jeddah, 21478 Saudi Arabia; 10 Research Department, Psychiatric Hospital of the Cross, Jal Eddib, Lebanon; 11 Applied Science Research Center, Applied Science Private University, Amman, Jordan

**Keywords:** External shame, Internal shame, Psychometrics, Lebanon, Arabic

## Abstract

**Background:**

The concept of shame in Arab societies displays multiple differences when compared to Western societies in terms of nature, sources, types, and correlates. Surprisingly, we could not find any study investigating this increasingly important construct in Arab countries or the broad Arabic-speaking communities. This may likely be due to the lack of valid instruments assessing shame in the Arabic language. To address this major gap and contribute to the international literature, we sought to examine the psychometric properties of an Arabic translation of the External and Internal Shame Scale (EISS) among a community sample of Arabic-speaking adults from Lebanon.

**Methods:**

An online survey was conducted among Lebanese adults between July and August 2022. A total of 570 Lebanese adults completed the EISS, as well as Depression Anxiety Stress Scales, Other as shamer scale, and the Standardized Stigmatization Questionnaire. Exploratory-to-confirmatory (EFA-CFA) factor analyses were conducted.

**Results:**

Exploratory and confirmatory factor analyses supported a unidimensional model of EISS scores, with all eight items retained. Scores achieved scalar invariance across gender, with no significant difference reported between females and males. EISS scores were found to have adequate composite reliability (McDonald’s ω = 0.88 for the total score); as well as adequate patterns of correlations with depression, anxiety and stress symptoms, as well as stigmatization scores. Finally, our analyses provide support to the concurrent validity of the Arabic version of the scale, by showing that the EISS total scores strongly correlated with the external shame measure “other as shamer”.

**Conclusion:**

Although further validations are necessary before our findings could be generalized, we preliminarily suggest that this is a short, easy-to-use, self-report scale that enables a reliable and valid measure of the shame construct among Arabic-speaking people.

## Background

Shame is a “self-conscious” feeling affecting a person’s sense of self and wellbeing [1]. It has been suggested that shame develops as a mechanism to control psychobiological reactions to social status. Historically, researchers referred to shame and guilt interchangeably. Shame and guilt share several common characteristics, including the fact that both are self-conscious emotions induced by self-evaluation and self-reflection [2], both help with self-regulation [3], both generate feelings of distress [4]. In addition, both constructs often coexist [5] and correlate with each other [6–8]. Despite the similarities, major differences between shame and guilt also exist. One difference lies to motivational consequences, with shame being driven by defensive interpersonal detachment and separation; while guilt involves positive preventive incentives [9, 10]. As opposed to guilt proneness, shame proneness is linked to a broad range of psychosomatic manifestations, including depression, suicidality, low self-esteem, eating problems, and posttraumatic stress disorders [11]. Both constructs differentially correlate with empathy; with guilt relating positively with this construct and shame showing reversed patterns of association [10]. The two constructs also play distinct roles in behavioral problems, with shame positively correlating with illegal behavior and guilt inversely correlating with risky and antisocial behavior [12]. Therefore, using measures that differentiate between these behavioral and emotional aspects of shame and guilt, or that focus solely on each of these constructs, are necessary [11].

The biopsychosocial and evolutionary model claimed that two distinct types of the shame experience exist: “internal shame” (IS) and “external shame” (ES) [13, 14]. IS is related to internal dynamics of the self and refers to the poor opinion of the self (such as being empty, undesirable, isolated, inferior, or inadequate) [14]; and how the self-judges and feels about itself [15]. In the IS, there is a negative self-assessment of one’s emotions, attributes, and behavior, by focusing on the self’s imperfections and flaws [16, 17]. On the other hand, ES focuses on the social environment and the perception of being judged, attacked or rejected by others, or that others have bad impressions of oneself, such as being inferior, defective, unattractive, undesirable, or worthless [3, 14]. The two concepts of IS and ES have been shown to be highly correlated and interdependent; with individuals who experience IS being highly likely to also feel ES [18]. Both ES and IS can lead to social exclusion and regarded as different dimensions of the identical emotional experience. Both components of shame are closely linked and encompass the same core domains of inferiority/inadequacy, exclusion, emptiness, and criticism [19]. Both aspects of shame are furthermore crucial for social functioning and regarded as a sign or warning of possible social harm [19]. In this regard, prior research demonstrated that shame positively correlates with a range of behavioral and psychopathological indicators, including depressive symptoms [20], social anxiety [21, 22], other anxiety disorders symptoms [23, 24], disordered eating [21, 25], substance use [26], and suicidal behavior [27]. It is of note, however, that despite presenting a negative valence, the sense of shame may be adaptive, enabling a certain compliance with social and moral norms, and facilitating social interactions [3, 28]. Given that shame is associated with psychological conditions, assessing shame and validating scales to measure the shame construct is extremely relevant [29].

The measures that have been designed and largely used to assess shame were either targeting ES (e.g., the Other as Shamer Scale [18]), or IS (e.g., the Internalized shame scale [30]). It is only recently that a self-report measure assessing ES and IS simultaneously, and yielding a global score of sense of shame, has been developed by Ferreira et al. (i.e., the External and Internal Shame Scale (EISS) [19]). The EISS consists of 8 items assessing both ES and IS as well as shame as a general concept [19]. The original validation of the EISS in Portuguese among community adults showed good concurrent validity, internal consistency, and positive correlations with depressive symptoms [19]. Since then, the EISS has been translated in different cultures and languages other than English (e.g., Japanese [31], Portuguese and French [32]), it has also been adapted and validated for various populations (e.g., adolescents [1]). More recently, Matos et al. [32] provided evidence of the validity of the EISS in a cross-national study including community samples of adults from five countries (Portugal, France, Australia, Singapore and Japan). However, no Arabic version exists yet, to the best of our knowledge.

Although the shame construct represents a universal and basic human emotion [33–35], it has also been shown to vary in meaning and positioning across people and cultures [36]. Some societies are much more shaming than others (e.g., Japanese [37]). Shame would be more prominent in collectivistic cultures [38]. Regarding Arab societies in particular, the concept of shame displays multiple differences when compared to Western societies, including possible sources (e.g., meeting strangers), types (e.g., praised or respect-related shame, such as Arab children who are taught to be shy and respectful to others), and correlates (e.g., age- and sex-graded shame, such as speaking loudly in public which is shaming for Arab women but not for men) [39]. To date, there is still a very limited body of research investigating shame in Arab countries or the broad Arabic-speaking communities. This may likely be due to the lack of valid instruments assessing this concept in the Arabic language. For instance, a cross-cultural study by Grey et al. [40] revealed that Arab Emirati students displayed significantly greater tendency to evaluate the self negatively following an experience of shame than their Irish counterparts; and that, in contrast, Irish students reported higher levels of shame withdrawal following a transgression. In light of their results, authors suggested that shame would help Emirati individuals abide by the norms of the Muslim culture, and thus appears to play a regulatory function [41]; whereas shame seems to be related to a more maladaptive behavioral tendency (i.e., avoidance) in Irish individuals [40]. These differences in features of shame across the two countries have been explained by cultural variations in shame’s responses that extend beyond the dichotomous categorization of individualistic versus collectivistic cultures [40]. However, the scarcity of research in this area hampers our understanding of the mechanisms underlying the cultural diversity regarding the shame construct. Arab countries are becoming increasingly open to Westernization and globalization, and most of the Arab societies are experiencing continuous changes, making unclear whether concepts such shame are also changing. This highlight the urgent need for future research on this topic in Arab countries. To address this major gap, and contribute to the international literature in this increasingly important area, we sought to examine the psychometric properties of an Arabic translation of the EISS among a community sample of Arabic-speaking adults from Lebanon. We hypothesized that analyses will confirm the factor structure found in the original validation. We also expect that the Arabic EISS will show adequate reliability and validity.

## Methods

### Participants

A total of 570 Lebanese adults enrolled in this study, with a mean age of 24.59 years (*SD* = 6.75) and 68.6% women. Other sample characteristics are displayed in Table 1.

**Table 1:** Sociodemographic characteristics of the participants

Variable	Total sample (*N* = 570)	First split-half subsample (*n* = 277)	Second split-half subsample *(n* = 293)
Gender			
Men	179 (31.4%)	90 (32.5%)	89 (30.4%)
Women	391 (68.6%)	187 (67.5%)	204 (69.6%)
Marital status			
Single	477 (83.7%)	232 (83.8%)	245 (83.6%)
Married	93 (16.3%)	45 (16.2%)	48 (16.4%)
Education			
Secondary or less	29 (5.1%)	18 (6.5%)	11 (3.8%)
University	541 (94.9%)	259 (93.5%)	282 (96.2%)
Region of living			
Urban	280 (49.1%)	141 (50.9%)	139 (47.4%)
Rural	290 (50.9%)	136 (49.1%)	154 (52.6%)
	**Mean ± SD**		
Age (in years)	24.59 ± 6.75	24.88 ± 7.76	24.32 ± 5.63
Household crowding index (person/room)	1.10 ± 0.51	1.13 ± 0.56	1.07 ± 0.45

### Measures

*The External and Internal Shame Scale (EISS).* This scale consists of eight items, generated to measure the four central domains of general feelings of shame, and present in both ES and IS: inferiority/inadequacy, sense of exclusion, uselessness/emptiness and criticism/judgment [19]. Each of the dimensions is composed of four items: external (e.g., “I feel that others see me as uninteresting”) and internal dimensions (e.g., “I feel that I am different and inferior to the others”), to which the participants must answer using a 5-point scale (0 = “Never” to 4 = “Always”). Scores vary between 0 and 32 points, with higher values indicating higher global sense of shame.

#### Translation procedure

A common procedure of back-translation was followed in the present study for all non-validated scales, in which a text is translated from a source into a target language, and then independently back-translated into the source language by a second interpreter. Therefore, the English version of the EISS was translated to Arabic by a Lebanese translator who was completely unrelated to the study. Afterwards, a Lebanese psychologist with a full working proficiency in English, translated the Arabic version back to English. To evaluate the accuracy of the translation, the initial and back-translated English versions were compared [42, 43]; and any inconsistencies were detected and eliminated by a committee composed of the research team and the two translators. A pilot study was done on 20 participants to make sure that the questions are well understood; no changes were done afterwards.

*Other as shamer (OAS-2)* [44], a short version of the OAS [18], includes 8 items intended to measure external shame (global judgements of how people think others view them). Respondents are asked to rate on a 5-point scale (0–4) the frequency of their feelings and experiences in items such as “People distance themselves from me when I make mistakes”. Higher scores reveal high external shame.

*The* Standardized Stigmatization Questionnaire (SSQ) [45]: This is a 13-item, 4-point Likert measure assessing the perception of social stigmatization and the predisposition to enact stigmatization through three dimensions: Social self-interest, Evolutionary self-interest, and Psychological self-interest. More elevated scores refer to greater stigmatization.

*Depression Anxiety Stress Scale (DASS-8)*, validated in Arabic [46], comprises eight items, in three subscales: depression (three items e.g., *felt down hearted and blue*), anxiety (three items e.g., *felt scared without reason*), and stress (two items e.g., *was using a lot of my mental energy*) [46]. The total scores of the DASS-8 and its subscales range between 0 and 24, 0 to 9, 0 to 9, and 0 to 6, respectively.

*Demographics.* Participants were asked to provide their demographic details consisting of age, gender, highest educational attainment, region of living, marital status. The number of persons and rooms in the house were used to compute the household crowding index (person/room); the higher the number, the lower the socioeconomic status [47].

### Procedures

All data were collected via a Google Form link, between July and August 2022. The project was advertised on social media and needed between 10 and 15 min to be completed. The link was shared among participants and sent to all districts/governorates of Lebanon (Beirut, Mount Lebanon, North Lebanon, South Lebanon, and Bekaa) through social networks, using the snowball technique. Inclusion criteria for participation included being of a resident and citizen of Lebanon of adult age. Internet protocol (IP) addresses were examined to ensure that no participant took the survey more than once. After providing digital informed consent, participants were asked to complete the instruments described above, which were presented in a pre-randomized order to control for order effects. The survey was anonymous and participants completed the survey voluntarily and without remuneration.

### Analytic strategy

#### Data treatment.

There were no missing responses in the dataset. To achieve the goal of this study, we first sought to identify the appropriate factor structure of scores on the Arabic EISS. Thus, we followed best-practice recommendations in adopting an EFA-to-CFA strategy [48, 49], which allows to examine the most suitable model of EISS scores in our sample without modelling limitations (i.e., through EFA) and to cross-validate the EFA-derived model, as well as the original unidimensional model (if discrepant), in a separate subsample (i.e., using CFA). To ensure adequate sample sizes for both EFA and CFA, we split the main sample using an SPSS computer-generated random technique; sample characteristics of the two split-halves are reported in Table 1. There were no significant differences between the two subsamples in terms of mean age, *t*(568) = 0.984, *p* = .326, household crowding index, *t*(568) = 0.072, *p* = .170, and the distribution of women and men, χ^2^(1) = 0.296, *p* = .587, single and married, χ^2^(1) = 0.002, *p* = .965, education level χ^2^(1) = 2.22, *p* = .136 and region of living χ^2^(1) = 0.683, *p* = .409.

#### Exploratory factor analysis.

To explore the factor structure of the EISS scale, we computed a principal-axis EFA with the first split-half subsample using the FACTOR software [50, 51]. We verified all requirements related to item-communality [52], average item correlations, and item-total correlations [53]. The Kaiser-Meyer-Olkin (KMO) measure of sampling adequacy (which should ideally be ≥ 0.80) and Bartlett’s test of sphericity (which should be significant) ensured the adequacy of our sample [54]. The procedure for determining the number of factors to extract was parallel analysis (PA; [55] using the Pearson correlation matrix. Weighted Root Mean Square Residual (WRMR) was also calculated to assess the model fit (values < 1 have been recommended to represent good fit) [56].

Item retention was based on the recommendation that items with “fair” loadings (i.e., ≥ 0.40), communality (i.e., ≥ 0.30) and above (i.e., ≥ 0.33) and with low inter-item correlations (suggestive of low item redundancy) as indicated by the anti-image correlation matrix should be retained [57].

#### Confirmatory factor analysis.

We used data from the second split-half to conduct a CFA using the SPSS AMOS v.29 software. A previous study suggested that the minimum sample size to conduct a confirmatory factor analysis ranges from 3 to 20 times the number of the scale’s variables [58]. Therefore, we assumed a minimum sample of 250 participants needed to have enough statistical power based on a ratio of 15 participants per one item of the scale, which was exceeded in this subsample. Parameter estimates were obtained using the robust maximum likelihood method and fit indices. Values ≤ 5 for χ²/df, and ≤ 0.08 for RMSEA, and 0.90 for CFI and TLI indicate good fit of the model to the data [59]. However, these cut-off values should not be interpreted rigidly (Heene, Hilbert, Draxler, Ziegler, & Bühner, 2011; Perry, Nicholls, Clough, & Crust, 2015); values between 0.08 and 0.10 for RMSEA can indicate acceptable but mediocre fit to the data [60, 61].

#### Gender invariance.

To examine gender invariance of the EISS, we conducted multi-group CFA [62] using the second split-half subsample. Measurement invariance was assessed at the configural, metric, and scalar levels [63]. Configural invariance implies that the latent scales’ variable(s) and the pattern of loadings of the latent variable(s) on indicators are similar across gender (i.e., the unconstrained latent model should fit the data well in both groups). Metric invariance implies that the magnitude of the loadings is similar across gender; this is tested by comparing two nested models consisting of a baseline model and an invariance model. Lastly, scalar invariance implies that both the item loadings and item intercepts are similar across gender and is examined using the same nested-model comparison strategy as with metric invariance [62]. Following previous recommendations [62, 64], we accepted ΔCFI ≤ 0.010 and ΔRMSEA ≤ 0.015 or ΔSRMR ≤ 0.010 (0.030 for factorial invariance) as evidence of invariance. We aimed to test for gender differences on latent EISS scores using an independent-samples *t*-test only if scalar or partial scalar invariance were established [65].

#### Further analyses.

Composite reliability in both subsamples was assessed using McDonald’s (1970) ω and its associated 95% CI, with values greater than 0.70 reflecting adequate composite reliability [66]. McDonald’s ω was selected as a measure of composite reliability because of known problems with the use of Cronbach’s α (e.g., [67]. To assess convergent and criterion-related validity, we examined bivariate correlations between the EISS scores and the additional measures included in the survey (DASS-8 and Others as shame). All scores had a normal distribution, as identified by skewness and kurtosis values varying between − 1 and + 1 [68]; therefore, Pearson correlation test was used to correlate two continuous variables, whereas the Student t test was used for the comparison of two means. Based on [69], values ≤ 0.10 were considered weak, ~ 0.30 were considered moderate, and ~ 0.50 were considered strong correlations.

## Results

### Exploratory factor analysis of the EISS

#### **Factor analysis on sample 1 (EISS- 8 items).**

The Bartlett’s test of sphericity, χ^2^(28) = 1190.2, *p* < .001, and KMO (0.892) indicated that the EISS items had adequate common variance for factor analysis. The results of the EFA revealed one factor, which explained 57.85% of the common variance. The WRMR value was also adequate (= 0.116; 95% CI 0.089-0.134), indicating good fit of the model. McDonald’s ω was adequate in the total subsample (ω = 0.89).

#### **Factor structure congruence and composite reliability (EISS- 8 items).**

 The factor loadings reported in Table 2. McDonald’s ω was adequate in women (ω = 0.88), men (ω = 0.89), and the total subsample (ω = 0.89).

### Confirmatory factor analysis of the EISS scale on sample 2

CFA indicated that fit indices of the 1-factor model of EISS (8 items) were acceptable: χ^2^/df = 66.71/18 = 3.70, RMSEA = 0.096 (90% CI 0.072, 0.122), SRMR = 0.037, CFI = 0.958, TLI = 0.935. When adding a correlation between items 2–3 and 4–7, the fit indices improved as follows: χ2/df = 66.71/18 = 3.71, RMSEA = 0.096 (90% CI 0.072, 0.122), SRMR = 0.036, CFI = 0.958, TLI = 0.935. The standardized estimates of factor loadings were all adequate (see Table 1).

#### **Composite reliability.**

Composite reliability of scores was adequate in women (ω = 0.88), men (ω = 0.90), and the total sample (ω = 0.88).

**Table 2:** *Items of the EISS in English and Factor Loadings Derived from the Exploratory Factor Analyses (EFA) in the First Split-Half Subsample, and Standardised Estimates of Factor Loadings from the Confirmatory Factor Analysis (CFA) in the Second Split-Half Subsample*

	EFA	CFA
Item		
1. Other people see me as not being up to their standards	0.76	0.77
2. I am isolated	0.77	0.73
3. Other people don’t understand me	0.74	0.65
4. I am different and inferior to others	0.81	0.67
5. Others are judgmental and critical of me	0.82	0.78
6. Other people see me as uninteresting	0.83	0.77
7. I am unworthy as a person	0.75	0.66
8. I am judgmental and critical of myself	0.58	0.54

### Gender invariance of the EISS 8 items scale

As reported in Table 3, all indices suggested that configural, metric, and scalar invariance was supported across gender. No significant difference was found between women (*M* = 7.67, *SD* = 5.76) and men (*M* = 8.92, *SD* = 6.54) in the second subsample, *t*(291) = 1.638, *p* = .102, d = 0.202.

**Table 3:** *Measurement Invariance of the EISS 8 items Across Gender in the Second Split-Half Subsample*

Model	χ²	*df*	CFI	RMSEA	SRMR	Model Comparison	Δχ²	ΔCFI	ΔRMSEA	ΔSRMR	Δ*df*	*p*
Configural	86.62	36	0.956	0.070	0.048							
Metric	91.49	43	0.958	0.062	0.053	Configural vs. metric	4.87	0.002	0.008	0.005	7	0.675
Scalar	100.76	50	0.956	0.059	0.053	Metric vs. scalar	9.27	0.002	0.003	< 0.001	7	0.233

*Note.* CFI = Comparative fit index; RMSEA = Steiger-Lind root mean square error of approximation; SRMR = Standardised root mean square residual.

### Convergent and Criterion-Related Validity

To assess the validity of the EISS scores, we examined bivariate correlations with all other measures included in the present study using the total sample. Higher EISS scores correlated significantly and positively with higher stigmatization dimensions (social self-interest, evolutionary self-interest, psychological self-interest), stress, depression, anxiety and other as shamer scores (Table 4).

**Table 4:** *Correlations of the EISS scores with the other measures in the second split-half subsample*

	1	2	3	4	5	6	7	8	9	10
1. EISS	1									
2. Social self-interest	0.23***	1								
3. Evolutionary self-interest	0.24***	0.64***	1							
4. Psychological self-interest	0.16**	0.53***	0.30***	1						
5. Stress	0.27***	0.08	0.10	0.08	1					
6. Depression	0.36***	0.17**	0.23***	0.22***	0.69***	1				
7. Anxiety	0.31***	0.14*	0.10	0.24***	0.63***	0.73***	1			
8. Other as shamer	0.80***	0.28***	0.25***	0.24***	0.21***	0.34***	0.31***	1		
9. Age	0.02	0.02	0.03	− 0.02	− 0.03	− 0.04	− 0.09	− 0.001	1	
10. Household crowding index	0.05	0.09	0.09	0.09	0.06	0.06	0.05	0.03	0.01	1

*p < .05; ** *p* < .01; *** *p* < .001

## Discussion

To date, there is only one measure that assesses both ES and IS as conceptualized by the evolutionary biopsychosocial model, the EISS. We aimed through the present study to validate this instrument in Arabic, in order to enable its use for clinical and research purposes among the wide Arabic-speaking populations globally. Our results support the validity of the EISS, as well as invariance across gender and composite reliability (McDonald’s ω = 0.88 for the total score). We suggest, accordingly, that the Arabic EISS is psychometrically sound and recommend its use for measuring shame in different Arab settings and contexts.

In terms of the factorial validity of the Arabic EISS, our analyses failed to support the two-factor structure originally supported by Ferreira et al. [19]. The eight items retained in the final model were based on a unidimensional factor structure, enabling the simultaneous assessment of ES and IS through a global score rather than two separate dimensions (ES and IS) as suggested by the developers of the scale. Indeed, fit of the unidimensional model of EISS scores in the present study was adequate when tested using both EFA and CFA. These results are broadly in line with the Japanese validation study in which EFA revealed that, unlike the originally proposed factor structure, all the eight items retained loaded into three factors [31]. This might be explained by cultural considerations. The EISS has been developed in a Western cultural background. As previously mentioned, the concept of shame as experienced by Arab people has its peculiarities and characteristics that differentiate it from that perceived by people from the Western, individualistic and developed countries. One important difference is that shame in the Arab culture is closely related to the fact that one imagines or knows that other people are watching them or thinking about them [39]. In other words, feeling IS would rather be intimately related to ES, and cannot exist without others’ perceptions about the self in Arab cultures. This could explain the unidimensionality of the shame concept in our sample. However, in light of these inconsistencies regarding the factor structure of the EISS, additional research still needs to be done to confirm the plausibility of the Arabic EISS structure in other Arab cultures, as well as in non-clinical samples. Our results also indicated that the single-factor structure of EISS scores was identical across women and men (in our EFA) and achieved full invariance (in our CFA). Previous studies have showed that variances of EISS scores exist across gender where females generally report higher scores both in ES and IS [19, 28, 70]. Similar patterns of comparisons of shame across gender has also been reported in the original validation [19], and in the adolescent version of the scale [1].

Our results showed that higher EISS scores were correlated with greater symptoms of depression, anxiety and stress. These findings are consistent with previous literature where the crucial role of shame in a range of psychological problems, including as depression, anxiety and stress, has been largely highlighted [29, 31, 71–73]. Overall, these results are consistent with the original validation by Ferreira et al. [19], and with those in adolescent samples [1]. Shame plays a critical role in the vulnerability and maintenance of mental health problems and psychological disorders, mainly depression [19]. Our findings also revealed that shame is associated with the three stigmatization dimensions (evolutionary, social, and psychological self-interest), which is consistent with previous literature data widely acknowledging a positive association between stigma and shame [74]. Stigma is found to result in numerous negative self-conscious emotions, including shame [74]. Finally, our analyses provide support to the concurrent validity of the Arabic version of the scale, by showing that the EISS total scores strongly correlated with the ES measure “other as shamer”. This demonstrates that the Arabic EISS is a valid scale to assess the shame construct.

### Limitations

A number of limitations of the present study could be improved in future studies. First, given the method of recruitment, which was performed online, and mostly attracted educated and female participants, it is unlikely that our sample is representative of the wider Lebanese population. Consequently, the gender invariance results should be interpreted with caution because of the numbers inequality between males and females. The response rate could not be determined. A further limitation of the present work was that we did not assess the relationship between shame and other relevant indices, such as other self-conscious emotions (e.g., guilt and humiliation) or psychological adjustment (e.g., self-reassurance). In addition, other psychometric characteristics, such as temporal stability and validity across Arab countries, still need to be examined to confirm the robustness of the Arabic EISS. Finally, psychometric properties of the Arabic version of the EISS have been examined in one Arab country, Lebanon; which may limit the generalizability of our findings to Arabic-speaking populations from other countries and cultural backgrounds. We highlight, however, that the scale was translated to literary Arabic and not Lebanese dialect; which guarantees its readability across all Arab countries. Nevertheless, we recognize that future linguistic invariance studies still need to be performed to further support the psychometric properties of the Arabic EISS. before generalizing the EISS to other Arab countries.

### Clinical implications

As for clinical implications, our findings point to the clinical relevance of the shame construct in Arab individuals. Indeed, the association of shame with high levels of psychological distress and stigma supports the previous assumptions that shame could be an indicator of a maladaptive response styles in Arab cultural backgrounds [40]. As such, professionals working in Arab settings ought to acknowledge the role of shame in mental health. Our findings shed light on the scarcity of research in this area in Arab contexts; which is partly due to a lack of universal, standardized and valid instruments to assess this construct. We therefore call for further studies to investigate the cultural peculiarities of shame and how it interferes with health indicators in Arabic-speaking communities. We believe that making the Arabic EISS available will hopefully encourage fruitful research output on shame from diverse disciplinary backgrounds, and have significant implications for improving the quality of cross-cultural studies in this area. Additionally, by ascertaining the consistency of measurement quality across gender groups, we suggest that the Arabic EISS can be used for gender comparisons in future research among Arabic-speaking populations.

## Conclusion

The present results support the reliability and validity of the one-factor, 7-item Arabic EISS. Although further validations are necessary before our findings could be generalized, we preliminarily suggest that this is a short, easy-to-use, self-report scale that enables a reliable and valid measure of the shame construct among Arabic-speaking people. The availability of the Arabic EISS will hopefully benefit researchers and clinicians who work in Arab settings, and allow for future cross-national comparisons of shame while including Arab cultures.

## Data Availability

All data generated or analysed during this study are not publicly available due the restrictions by the ethics committee. The dataset supporting the conclusions is available upon request to the corresponding author S.H.
